# Austrian College Students’ Experiences With Digital Media Learning During the First COVID-19 Lockdown

**DOI:** 10.3389/fpsyg.2022.734138

**Published:** 2022-02-07

**Authors:** Carrie Kovacs, Tanja Jadin, Christina Ortner

**Affiliations:** Faculty of Informatics, Communications and Media, Institute of Communication and Knowledge Media, University of Applied Sciences Upper Austria, Hagenberg, Austria

**Keywords:** COVID-19, multimedia learning, digital media use, online learning, e-learning, interactive media, attitudes, online tools

## Abstract

In 2020, the COVID-19 pandemic forced many nations to shut-down schools and universities, catapulting teachers and students into a new, challenging situation of 100% distance learning. To explore how the shift to full distance learning represented a break with previous teaching, we asked Austrian students (*n* = 874, 65% female, 34% male) which digital media they used before and during the first Corona lockdown, as well as which tools they wanted to use in the future. Students additionally reported on their attitudes and experiences with online learning. Results showed that students used certain tools, such as video, audio, e-assessments, and web conferencing systems, much more often during lockdown than they had before. Their use of classic digital media, such as e-mail, social communication tools, such as chat or online forums, and other interactive tools, such as wikis or educational games, hardly changed at all. Their attitudes toward multimedia learning were positively related to their media use. In their open responses (*n* = 137), students identified advantages of online learning (flexibility and self-directed learning), as well as disadvantages (limited social interaction) and challenges (motivation and self-discipline). As a group, they also expressed a clear preference for a balanced combination of online- and offline teaching in the future. However, individual students did prefer fully online or offline learning modes, depending on their personal circumstances and educational goals. We view this as a call to researchers and educators alike to explore ways in which the advantages of online and face-to-face learning can best be combined to meet the changed needs and expectations of organizations, students, and teachers in a future “after Corona.”

## Introduction

In spring of 2020, the COVID-19 pandemic precipitated a widespread shut-down of public life throughout the world. Along with countless other public and private organizations, schools and universities found themselves in an unprecedented situation. Though countries all around the world were in similar circumstances, higher education institutions in different countries employed widely different response strategies (Crawford et al., 2020). In many countries, including Austria, universities suddenly closed, switching to a complete distance learning modus—generally using some form of online instruction—within a few days or even hours.

As might be expected, such an abrupt upheaval was not only challenging for instructors, but also for students. Lack of motivation, Internet problems, limited interaction among students and instructors, trouble concentrating, difficulty finding school-life balance, learning problems, and lack of support were identified as particularly important challenges in two early COVID-19 studies (Adnan and Anwar, 2020; Aguilera-Hermida, 2020). In addition to these problems, students reported deficiencies in the quality of online discussions and an absence of structure in online class settings (Nambiar, 2020). Despite such drawbacks, students also identified benefits in the new learning situation. US students, for instance, reported having more time for family, hobbies, self-care, and personal growth (Aguilera-Hermida, 2020). Indian students reported practical advantages, such as time saved commuting and having online videos of lectures to refer to after class (Nambiar, 2020). Different factors played a role in how positively students viewed the distance-learning situation. For instance, one study found that instructional quality played an important part in predicting students’ satisfaction with online learning (Gopal et al., 2021). Austrian and Finish students’ self-reported feelings of competence were the strongest predictor for positive emotions during online learning, though both competence and autonomy predicted intrinsic learning motivation (Holzer et al., 2021). In a further study looking at perceptions of online learning during the lockdown, Rizun and Strzelecki (2020) used data from 1,692 Polish participants to predict students’ acceptance of distance learning. They found that distance-learning enjoyment and self-efficacy were better predictors of acceptance than computer experience or anxiety, but also that perceived ease of use and perceived usefulness were key mediators between enjoyment/self-efficacy and overall attitudes toward and intention to use distance learning. Thus, pedagogical and psychological factors as well as specific technical aspects seem central in determining how university students perceive distance learning generally and online learning during the COVID-19 lockdown specifically.

Online learning itself is not new or unique to the COVID-19 pandemic. Before COVID-19, higher education institutions had long pursued various e-learning strategies, both in the form of fully online learning and in blended online/offline formats. E-learning has both advantages and disadvantages. On the one hand, online learning offers more flexibility in time and space, ease of access to a huge amount of information, different interaction possibilities, reduced costs (e.g., travel), lower barriers in initiating certain kinds of communication, and support of self-paced, individual learning (Arkorful and Abaidoo, 2014). Paechter and Maier (2010), investigating Austrian students’ preferences, found that online learning was considered better in offering clear content structure and supporting individual learning processes. Conversely, students favored face-to-face learning for communication and collaborative learning processes. A similar result was found among US students asked to explain either their satisfaction or dissatisfaction with online learning. Satisfied students most commonly cited convenience as the cause for their satisfaction; dissatisfied students most commonly cited lack of interaction and communication as reason for their dissatisfaction (Cole et al., 2014). Such reports of interactional deficits in online learning also fit with results showing that the “nature of e-learning”—including its impersonal nature and lack of interaction with other learners—was considered a central barrier to future online learning among a group of Australian employees (Becker et al., 2013). Similarly, both Taiwanese and American students rated face-to-face courses more positively than online courses in terms of communication/interaction (Young and Duncan, 2014; Bali and Liu, 2018). These findings all echo and support a more general claim that subjective feelings of social presence during online communication are an important predictor of student satisfaction and thus particularly relevant in computer-mediated learning contexts (e.g., Gunawardena, 1995; Lowenthal, 2010).

Given this evidence that limited social interaction is a challenge to successful online learning, a closer examination of how specific online media tools may facilitate communication processes seems merited. Hsieh and Cho (2011), for instance, argued that instructor-student interactive tools are more useful and satisfying to students than self-paced tools because they provide greater media richness, social presence, and thus greater information quality. These authors found correlational evidence among a group of Chinese students to support their claim that e-learning tools’ ability to facilitate social interaction is a key aspect driving students’ evaluations of those tools. A similar focus on interaction is also found in Anderson’s (2008) theoretical classification of educational media along two dimensions: the extent to which a medium can be used independently of time and distance, and the extent to which it supports interaction. Media such as television—or, more recently, online educational videos—can be used largely independently of time and place, but they allow for relatively little interaction. Face-to-face discussions show an opposite pattern, with low independence of time and place but high levels of interaction. Media such as video or audio conferencing fall somewhere in between. According to Anderson, successful online learning occurs when teachers are able to switch flexibly between appropriate media and communication forms for a given learning context.

As educators flexibly respond to students’ needs, new technologies and an ever-growing collection of readily available educational software have radically increased the size of the media toolbox from which they can draw. Web 2.0 and social media allow for flexible and spontaneous interaction inside and outside the classroom, falling fairly high on both the interaction and the independence dimension of Anderson’s model. In fact, e-learning with social media embedded in specific course design has been shown to facilitate knowledge acquisition and knowledge building (Mauss and Jadin, 2013; Mnkandla and Minnaar, 2017). Ubiquitous mobile devices have further increased learners’ independence of time and place, while simulation software and virtual reality technologies expand the bounds of feasible hands-on exercises. Besides supporting independence of time and place, such new tools are also often high in interactive potential, meaning that they offer ever more and easier technological possibilities for interactive learning. Even below any new cutting edge of technological developments, however, computer-supported collaborative learning has long been possible and didactically effective, as shown by a meta-analysis of 143 studies published between 2004 and 2014 (Jeong et al., 2019).

Despite this potential, interactive communication media have traditionally remained underused in tertiary education (OECD, 2005; Persike and Friedrich, 2016). For instance, Persike and Friedrich (2016) found that classic tools, such as e-mail, were still the dominant media used for online learning among a Germany-wide sample of 27,473 university students. More than 50% of students were classified either as “PDF-users” (employing primarily classic digital media, such as PDF-documents, e-mail, and presentation slides) or as “e-examinees” (employing classic digital media and e-assessments only). While a further 22% were “video-learners,” who reported high usage of classic and audiovisual media, only 21% of students could be classified as “digital all-rounders” employing a wide variety of digital media in their studies, including interactive media. This is in line with results of a much smaller Romanian study, which found that although students rated Web 2.0 and collaborative tools as helpful and useful for educational purposes, they used such tools primarily for finding information (e.g., on Wikipedia) and not as intended by the innovators in the sense of user generated content (Popescu, 2010). Effectively using Web 2.0 and other new technologies to facilitate collaborative learning and communication processes demands distinctive pedagogical approaches. Kreijns et al. (2003) argued that instructors must steer clear of two major pitfalls when implementing collaborative online learning: taking social interaction for granted and restricting social interaction to cognitive processes. In order to realize the full interactive potential of online communication media, they suggest instructors must actively promote “sociable” collaborative online learning environments through a variety of instructional strategies. This is in line with research on face-to-face education, which shows that effective collaborative learning depends in large part on appropriate instructor support (Webb, 2009). It follows that one of the reasons for underutilization of online communication media in the past has been that it was not accompanied by appropriate pedagogical strategies. In addition to pedagogical concerns, issues of workload, cost, inadequate infrastructure, and lack of technical support have also been named as barriers to online teaching in higher education settings (Keengwe and Kidd, 2010). Thus, the 2020 COVID-19 lockdown forced online learning on an educational community with some decades’ worth of e-learning experience in principal, but very limited e-learning prevalence in practice.

Such lack of e-learning experience can no longer be reasonably assumed; everyone in school systems subject to the lockdown gained experience with distance learning in 2020, generally through some form of online instruction. Teachers (Nambiar, 2020) as well as students (Rahiem, 2020) reported using a variety of different online tools. The current study expands on this research by exploring which types of tools specifically “boomed” in Austrian higher education institutions during the first COVID-19 lockdown. In addition to asking students which specific media they used for educational purposes during the lockdown, we also questioned them about their former usage habits (“before Corona”) and about their desired usage in a future “after Corona.” Purpose of this explorative quantitative survey was (1) to get a general impression of usage and changes in usage of specific media types during the first Corona lockdown and (2) to determine how media usage (and desired usage) related to students’ overall attitudes toward multimedia learning. As part of our first broad research question, we also explored whether and in what proportions we could identify individuals corresponding to the four media user types of Persike and Friedrich’s (2016) categorization based on students’ “before Corona” media usage reports. Assuming that we could find similar media user types, we were interested in discovering how these groups’ media use developed during the lockdown. Due to the large number of rather elaborated answers to an open-ended question at the end of the questionnaire, we were unexpectedly able to extend our explorative quantitative methodology into a mixed-methods approach. Students’ spontaneous statements allowed us (3) to explore what they saw as central differences between online and offline learning, what conditions for successful online learning they identified, and what they recommended for future online practice in higher education.

## Materials and Methods

### Study Design and Recruitment

In order to explore students’ use of educational media before and during the lockdown, we drew upon a correlational, cross-sectional, and largely quantitative survey design. We subsequently extended our study by a qualitative analysis of written comments left by participants at the end of the survey, resulting in an explorative mixed-methods approach. The study was planned and conducted with the help of 16 students fulfilling a research course requirement in their interdisciplinary Bachelor’s degree program. In April and May of 2020, we distributed an online questionnaire to representatives of all 96 public and private universities located in Austria at that time. In addition to using official contact e-mail addresses obtained from the websites of these institutions, students also used their private connections to student representative groups and members of individual study programs to recruit participants. Through this combination of systematic and convenience sampling, we were able to recruit an initial 1,514 hits on the first page of the online questionnaire. Of these initial visitors to the instruction page, 1100 (73%) gave their consent to participate in the study. Only those 1,037 (68%) participants who reported being current university students were asked to continue with the survey; this number was further reduced to 1,033 students who reported attending an Austrian (as opposed to German) school. The sample decreased to 874 (58%) through a control question halfway through the survey, which instructed participants to select a specific answer option. Of these 874 students, 132 provided comments about e-learning at the end of the survey. Five additional students left such comments after having skipped or incorrectly answered the control question. Though careless responding seemed likely for these students’ rating scale data and justified its continued omission from the quantitative analysis, their open responses were plausible and relevant. Thus, we chose to include these comments in the qualitative analysis. This resulted in a final qualitative sample of 137 students.

### Measures

In addition to basic information about their age, gender, course of studies, and home institution, students were asked to estimate the intensity of their use of specific digital media for educational purposes before and during the first COVID-19 lockdown (March 2020), their preferred intensity of such media use in the future, as well as their overall attitudes toward multimedia learning. At the end of the survey, they were asked whether they had any further comments and provided with an open text input field.

*Media use* items were adapted from a publically funded German educational research study conducted by Persike and Friedrich in 2016. The original study asked students to report whether they used 20 specific digital media types in the course of their studies. These media were grouped into five broad categories: (1) *classic digital media* (e.g., digital texts and e-mail), (2) *social communication tools* (e.g., chat and forums), (3) *e-exams* (e-assessments and e-exams), (4) *audiovisual media* (audio, video, and web-based tutorials), and (5) *interactive tools* (e.g., educational games, and online office tools). Though we largely adopted this list and categorization, we changed the survey instructions to ask students how intensely they had used the given media for purposes related to their studies (a) before the lockdown and (b) during the lockdown, as well as (c) how intensely they hoped to use these media in the future “after Corona.” We changed the original 4-point categorical response scale to an ordered 6-point scale with the categories *very intensely* (6, “sehr intensiv”), *fairly intensely* (5, “ziemlich intensiv”), *intensely* (4, “intensiv”), *moderately* (3, “mäßig”), *slightly* (2, “wenig”), and *not at all* (1, “gar nicht”). Participants were additionally provided with a *do not know* (“weiß nicht”) answer option, which was treated as a missing value in subsequent analyses. Based on qualitative feedback from the 16 students involved in survey construction and a small-scale pretest (*n* < 10), we also changed the original items slightly to clarify their meaning. On the one hand, we added concrete examples to three media descriptions—for instance, “Chat” became “Chat/Messenger (e.g., WhatsApp, Slack).” Additionally, because students had trouble differentiating between the items “E-Assessments” and “E-Exams,” we combined them to form a single item “E-Assessments/E-Exams” (this meant that the final media category *e-exams* consisted of only one item). To form our media usage indices, we calculated the mean self-reported usage intensity for each of the five broad media categories; this made it possible to perform cluster analyses conceptually comparable to those of Persike and Friedrich (2016).

*Attitude toward multimedia learning* was measured using a 10-item instrument developed by Tigges (2008). Participants were asked to rate their agreement on a 4-point scale with the answer options *agree* (4, “stimme voll zu”), *somewhat agree* (3, “stimme eher zu”), *somewhat disagree* (2, “stimme eher nicht zu”), and *disagree* (1, “stimme nicht zu”). Seven of the 10 items indicated positive attitudes toward multimedia learning (e.g., “Multimedia increases motivation.”) while three reverse-coded items indicated negative attitudes (e.g., “Virtual instruction makes people lonely.”). Two items made obsolete by the current lockdown (e.g., “I think it would be good to be able to take part in online classes from home and not have to come to the university as often.”) were adapted to refer to a future after the lockdown (e.g., “Even after the Corona crisis is over, I think it would be good […]”).

Additionally, students’ *appraisal of online learning* in their open comments was rated in terms of valence by two authors of the study using inductive structuring qualitative content analysis according to Mayring (2014). Using an open coding approach based on initial perusal of the data, we developed category definitions, anchor examples, and coding rules to classify students’ appraisals of online learning. We then applied these coding rules to rate individual comments as either *negative* (−1), *neutral* (0), or *positive* (+1). This rating was applied to each comment as a whole, resulting in an *overall valence of open response* rating. Because students’ appraisals of online learning in general often explicitly contradicted their appraisals of their experienced implementation of online learning during the lockdown, we additionally used the same category levels to rate students’ general *appraisal of online learning* as well as their *appraisal of online learning implementation* separately. We felt this to be an interesting distinction in understanding attitudes toward online learning, especially negative or ambivalent attitudes, since it revealed that these could be due more to problematic implementation than inherent objections to online learning (though experiences of concrete implementation presumably do impact abstract attitudes toward online learning and vice versa). If students made no general statements about online learning or no specific statements about online learning implementation, ratings for the given variable were coded as missing. For instance, the comment “Online learning is a great addition but should not be treated as a replacement” was given an overall neutral valence rating (0), since positive and limiting statements were roughly balanced. It also received neutral ranking as an appraisal of online learning but was coded as missing in terms of online learning implementation. In contrast, the statement “I think that many universities are way behind with digital media and also aren’t willing to switch to something new. I hope the current situation changes that!” was coded as having overall negative valence (−1), as constituting a negative appraisal of online learning implementation, and as including no appraisal of online learning in general. As mentioned above, only comments dealing in some way with online learning were included in the qualitative sample, so that all comments received an overall valence rating as well as a rating in at least one of the two coded subcategories. Overall valence ratings were independently coded by a second rater, showing substantial (Landis and Koch, 1977) interrater reliability (Cohen’s κ = 0.67).

### Sample Characteristics

Our final sample of 874 Austrian students consisted of 386 (44%) students from public universities, 385 (44%) students from applied universities, 65 (7%) students from private colleges, and 38 (4%) students from other tertiary education institutes (e.g., teacher training colleges and seminaries). Due to varying participation among individual institutions, the final sample was geographically skewed, with 344 (39%) students attending schools in Upper Austria, 175 (20%) in Lower Austria, 150 (17%) in Tyrol, 71 (8%) in Vienna, 55 (6%) in Styria, 41 (5%) in Burgenland, 33 (4%) in Salzburg, and only five students in Carinthia and Vorarlberg combined. About half of these students (*n* = 426, 49%) was pursuing a bachelor’s degree, 254 (29%) were in a master’s program, 128 (15%) were in the traditional Austrian diploma program which provides the equivalent of a bachelor’s and master’s degree when completed, 46 (5%) were pursuing a doctorate, and 20 (2%) students gave no response or reported being in other degree programs (e.g., medical degree). The majority of these students (*n* = 602, 69%) reported being part of full-time degree programs originally designed to consist primarily of face-to-face teaching; a further 214 (25%) students reported being in a part-time face-to-face program aimed at working students. Only 24 (3%) students reported having signed up for a part-time distance learning degree program, while six (1%) students reported being in a full-time distance learning program. A total 570 (65%) students identified as female, 296 (34%) as male, three students as diverse and five students gave no gender information. Participants’ ages ranged from 17 to 69, with a median of 24 years and a mean age of 27 years (*SD* = 8.8), though a substantial portion of the sample (*n* = 183, 21%) gave no age information.

A total of 137 (16%) students left comments at the end of the survey dealing in some way with e-learning or the distance learning situation, allowing them to be included in the qualitative analysis (19 additional comments were omitted from analysis because they referred to the survey itself or gave unrelated information about students’ personal situations). In order to identify any systematic differences between our full sample and the participants who left comments, we performed a drop-out analysis using logistic regression to predict inclusion in the qualitative analysis on the basis of gender, age, school location, type of school (university, applied university, etc.), type of degree program (bachelor’s, master’s, etc.), media usage during the lockdown, and attitudes toward multimedia learning. Due to the very small case numbers, we excluded the categories “diverse” and “no answer” from the variable gender, the states Carinthia and Vorarlberg from school locations and the categories “other” and “other private educational institution” from the variable school type. Results of the full logistic regression model showed a significant omnibus model test, *Χ*^2^(21) = 41.06, *p* = 0.006 with a Cox and Snell *R*^2^ of 0.06 and a Nagelkerke *R*^2^ of 0.11. An examination of individual predictors showed that age (*OR* = 1.03, *p* = 0.037), multimedia learning attitudes, (*OR* = 0.68, *p* = 0.026), as well as interactive (*OR* = 1.79, *p* = 0.005) and social communication (*OR* = 0.66, *p* = 0.009) tool usage during the first Corona lockdown reached statistical significance in predicting whether a participant commented or not. Commenting students were older (*M* = 29 years, *SD* = 10.5) than non-commenting students (*M* = 27 years, *SD* = 8.3) and reported less positive attitudes toward multimedia learning (*M* = 2.58, *SD* = 0.76) than non-commenting students (*M* = 2.70, *SD* = 0.65). They also reported slightly less intense use of social communication tools (*M* = 2.36, *SD* = 0.85) than non-commenting students (*M* = 2.58, *SD* = 0.95). Though the mean scores of interactive media use were descriptively equal for commenting and non-commenting students (*M* = 2.52, *SD* = 0.77), the odds ratio of 1.79 found in the regression analysis revealed that commenting students were actually likely to report more intense use of interactive media than non-commenting students when controlling for other relevant variables. Thus, our qualitative analyses (which reflect only the statements of commenting students) were not completely representative for our total sample: older participants with slightly less positive media attitudes and lower social communication tool usage (but higher interactive tool usage) seem to be somewhat overrepresented in relation to the total sample contributing to our quantitative results.

### Data Analyses

Quantitative analyses were performed using IBM SPSS Version 27 and JASP Version 0.10.2,^1^ graphs were created using Microsoft Excel and base R. Qualitative analyses were conducted with the help of MAXQDA 2018.

Students’ appraisals of online learning were quantified according to Mayring (2014), as described in the Measures section above. Additionally, a data-driven, category-based thematic qualitative content analysis was used to gain a deeper understanding of the full breadth of students’ open comments (see Kuckartz, 2019).

In order to determine overall changes in media use for individual types of media over time, we calculated a MANOVA with time (*before*, *during*, and *after*) as within-subject factor and usage of each of the 19 digital media as the outcome variables. Due to the explorative nature of the study and the large number of variables, we chose to analyze individual digital media usage ratings only descriptively on the basis of means and standard deviations instead of reporting all possible univariate and post-hoc tests. In general, because the assumptions for statistical inference (random sampling and independent observations) were not met by our data, all our inferential analyses should be seen as a type of small-scale data-mining with the aim of identifying possible effects of interest, not as stringent hypothesis tests.

In order to explore relationships between media use and attitudes, we aggregated the usage ratings for the 19 media types into the five mean usage indices described above (*classic digital media*, *social communication tools*, *e-exams*, *audiovisual media*, and *interactive tools*) for each assessed time point (before, during, and after the first Corona lockdown). Relationships between these media usage indices and overall *attitude toward multimedia learning* were calculated using Pearson’s correlation coefficients. Relationships between usage indices and *appraisals of online learning* gleaned from the open-ended questions were quantified using Kendall’s rank coefficient τ. The large number of relationships tested means that our correlational analyses likely suffer from alpha-inflation.

We also attempted to reproduce Persike and Friedrich’s (2016) media usage typology based on the “before Corona” media usage indices, since these seemed most likely to be comparable to the 2016 data and would thus allow us to see differences between these groups’ original media usage and their self-reported usage during the first Corona lockdown as well as their desired usage “after Corona.” We performed *k*-means cluster analysis on the five *z*-standardized usage indices, assuming a 4-cluster solution (based on the number of clusters found by Persike and Friedrich). We then performed a univariate ANOVA to ensure that resulting clusters differed in terms of the usage indices on which they had been based, and a discriminant analysis to cross-validate the classification of individuals to specific clusters (assuming marginal totals based on the observed frequency distribution among the four clusters). Note that this was not a direct replication of Persike and Friedrich’s analysis, since their original classification was based on dichotomous usage reports aggregated to percentage usage scores, not on mean usage indices. After forming the clusters, we tested association between cluster membership and attitudes through a univariate ANOVA with cluster (*PDF-user*, *video-learner*, *e-examinee*, and *digital all-rounder*) as between-subjects factor and *attitude toward multimedia learning* as the outcome variable.

## Results

### Media Use

**Figure 1:**
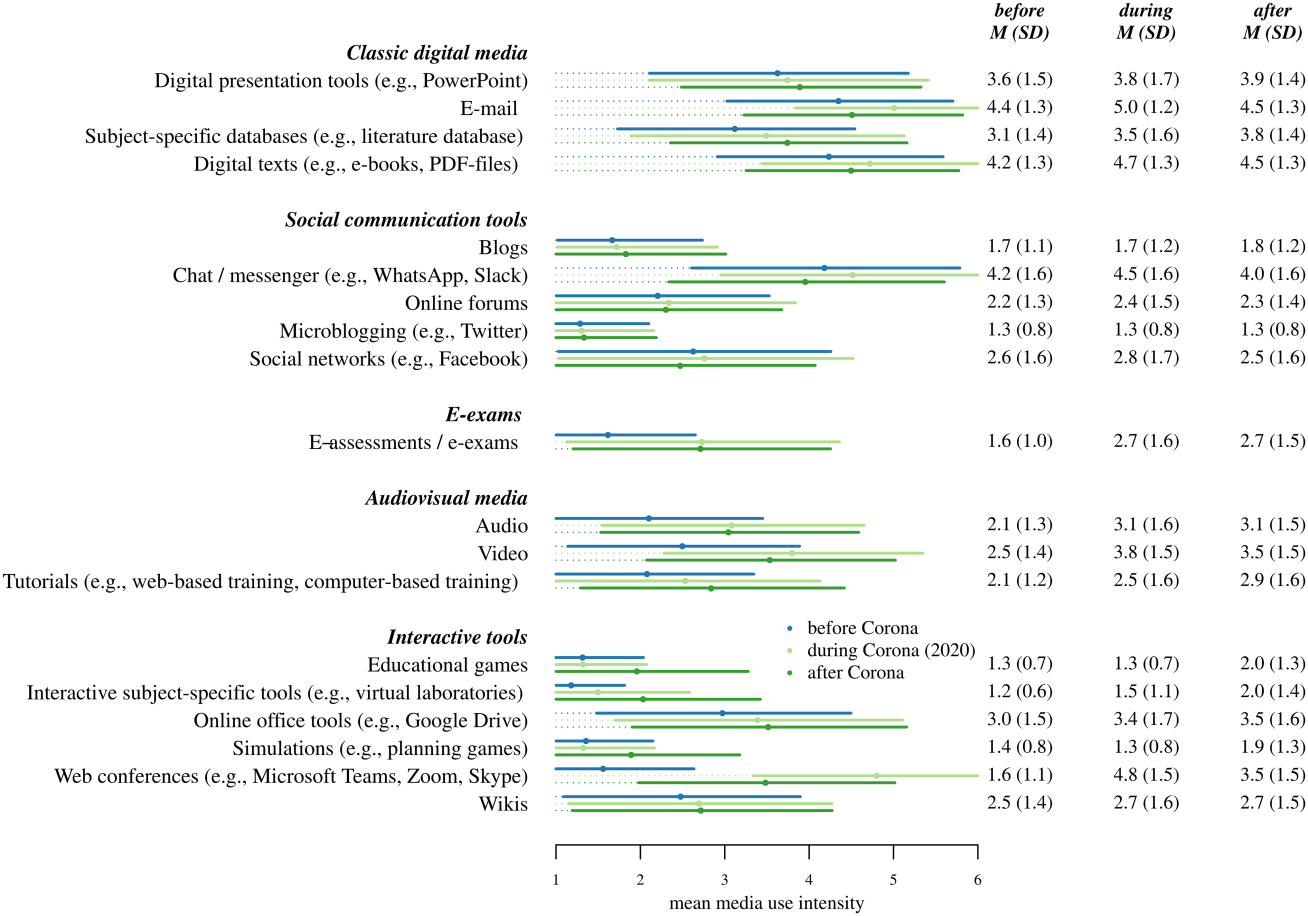
Self-reported intensity of media use *before* and *during* the first Corona lockdown, as well as intensity desired *after* Corona (751 < *n* < 873, error bars represent standard deviations, truncated where they exceed the limits of the response scale).

Students’ self-reported use of different digital media is shown in Figure 1. The MANOVA calculated across all of the 19 digital media types showed significant overall differences in usage across time, *F*(38,488) = 68.14, *p* < 0.001. *Classic digital media* use was reported as fairly high before the 2020 lockdown, with presentation tools, e-mail, databases, and digital texts all showing mean usage above the response scale midpoint (3, “moderately”). Among all other types of media, only chat/messenger (in the category *social communication tools*) reached similarly high usage levels, though online office tools (among the *interactive tools*) were used moderately before the lockdown. Use of all other tools was comparatively low. This profile changed substantially during the lockdown. There was a sharp rise in use of *audiovisual media* (e.g., video and online tutorials), and a particularly steep increase in the interactive tool web conferencing, which changed from a seldom-used tool to one of the most frequently used tools, second only to e-mail and comparable to digital texts or chat. Web conferencing also showed the largest absolute discrepancy between usage “during Corona” and desired usage “after Corona”—students reported a desire to use this medium much less intensely after the end of the lockdown. In contrast, many of the seldom-used interactive tools showed a discrepancy in the opposite direction: students wished these rare tools to be used a bit more intensely after Corona, though not as intensely as, for instance, audiovisual media or e-assessment. Students also, however, expressed quite a bit of uncertainty in these “wish” ratings. While the number of “do not know” answers per tool ranged between 0 and 46 (5%) for media use before and during the lockdown, individual tools like wikis and interactive subject-specific tools received up to 86 (10%) “do not know” ratings when participants were asked how often these should be used hypothetically in the future.

### Media Use Types

**Figure 2:**
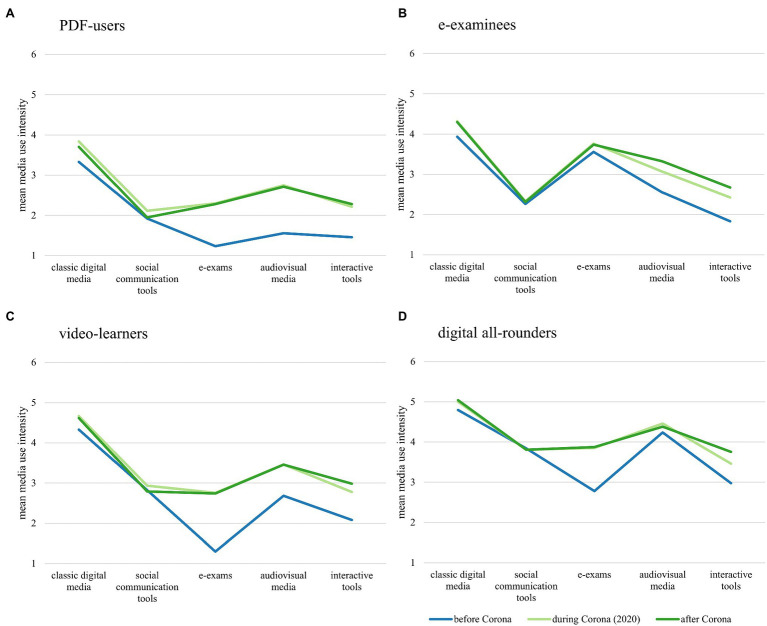
Students’ media usage profiles before and during the 2020 Corona lockdown as well as their desired use after Corona. Graphs **(A–D)** show data for each of the 4 media use clusters respectively (clusters based on self-reported media usage “before Corona”).

We were able to generate an acceptable 4-cluster media use typology based on the “before Corona” media usage indices. Univariate ANOVAs comparing the resulting clusters showed significant differences for all media usage indices (all *p* < 0.001), and a subsequent discriminant analysis resulted in correct re-classification of 97% of clustered cases (36% would be expected by chance based on the observed cluster distribution). Based on their pre-lockdown media usage, we were able to identify clusters corresponding roughly to the original typology. Of the 874 students classified, 418 (48%) could be called “PDF-users,” reporting a high percentage of classic digital media use and moderate to low use of all other media types. A second group of 96 (11%) “e-examinees” had a profile very similar to this first cluster, except that they reported high, rather than low, e-exam use. A large cluster of 289 (33%) students corresponded roughly to the original “video-learner” group, which was also similar to the “PDF-users” group except that students reported higher audiovisual and interactive media use. The smallest cluster was formed by 71 (8%) “digital all-rounders,” who reported consistently moderate to high use of all five media types, though their e-exam use was slightly lower than the “e-examinee” group. Figure 2 shows usage intensity profiles for each of the resulting clusters before and during the lockdown, as well as their desired usage “after Corona.” All groups showed an increase in use of e-exams, audiovisual media, and interactive tools during the first Corona lockdown, though this increase was smallest for those clusters with higher initial values (i.e., digital all-rounders and e-examinees). Though use of these three types of media did increase for “PDF-users,” this group maintained the lowest usage levels among the four clusters in their “during Corona” and “after Corona” values. Use of social communication tools showed almost no change for any of the clusters, while classic digital media increased only slightly. Desired media usage after Corona was virtually identical to usage during the first lockdown for all user groups and all types of media, with the exception of interactive tools: all four groups desired slightly less intense usage of these tools in the future.

### Attitudes Toward Multimedia and Online Learning

Comparing the four clusters in terms of attitudes toward multimedia learning showed a coherent picture: PDF-users reported the least positive attitudes (*M* = 2.5, *SD* = 0.67), digital all-rounders reported the most positive attitudes (*M* = 3.1, *SD* = 0.54), and e-examinees (*M* = 2.8, *SD* = 0.69) and video-learners (*M* = 2.8, *SD* = 0.64) fell between these two extremes, *F*(3,825) = 351.66, *p* < 0.001. This echoed the results of correlation analysis showing that multimedia attitudes related positively to all forms of media use, though this relationship was descriptively stronger when considering desired future media use (0.23 < *r* < 0.51) than actual use before or during the first Corona lockdown (0.09 < *r* < 0.23; see Table 1).

**Table 1:** Correlations between attitude toward multimedia learning and intensity of media use.

		(1)	(2)	(3)	(4)	(5)	(6)	(7)	(8)	(9)	(10)	(11)	(12)	(13)	(14)	(15)	(16)	(17)	(18)
**Media use before Corona**																																			
(1)	classic digital media	(0.66)																	
(2)	social communication tools	0.42^***^	(0.69)																
(3)	e-exams	0.19^***^	0.18^***^	—															
(4)	audiovisual media	0.30^***^	0.47^***^	0.30^***^	(0.78)														
(5)	interactive tools	0.37^***^	0.50^***^	0.25^***^	0.56^***^	(0.61)													
**Media use during Corona (2020)**																																			
(6)	classic digital media	0.68^***^	0.36^***^	0.14^***^	0.22^***^	0.32^***^	(0.69)												
(7)	social communication tools	0.34^***^	0.81^***^	0.12^***^	0.39^***^	0.42^***^	0.46^***^	(0.70)											
(8)	e-exams	0.19^***^	0.24^***^	0.39^***^	0.18^***^	0.23^***^	0.32^***^	0.28^***^	—										
(9)	audiovisual media	0.15^***^	0.32^***^	0.08^*^	0.46^***^	0.36^***^	0.33^***^	0.45^***^	0.41^***^	(0.74)									
(10)	interactive tools	0.25^***^	0.40^***^	0.11^**^	0.37^***^	0.64^***^	0.42^***^	0.50^***^	0.38^***^	0.58^***^	(0.67)								
**Desired media use after Corona**																																			
(11)	classic digital media	0.72^***^	0.41^***^	0.17 ^***^	0.30^***^	0.36^***^	0.73^***^	0.42^***^	0.25^***^	0.29^***^	0.36^***^	(0.73)							
(12)	social communication tools	0.35^***^	0.76^***^	0.16^***^	0.41^***^	0.45^***^	0.37^***^	0.81^***^	0.23^***^	0.40^***^	0.45^***^	0.49^***^	(0.72)						
(13)	e-exams	0.17^***^	0.21^***^	0.39^***^	0.19^***^	0.23^***^	0.21^***^	0.19^***^	0.38^***^	0.22^***^	0.22^***^	0.32^***^	0.28^***^	—					
(14)	audiovisual media	0.16^***^	0.24^***^	0.14^***^	0.45^***^	0.32^***^	0.24^***^	0.28^***^	0.20^***^	0.55^***^	0.39^***^	0.37^***^	0.40^***^	0.52^***^	(0.81)				
(15)	interactive tools	0.26^***^	0.27^***^	0.12^***^	0.35^***^	0.54^***^	0.30^***^	0.30^***^	0.23^***^	0.38^***^	0.57^***^	0.44^***^	0.43^***^	0.50^***^	0.65^***^	(0.80)			
**Attitudes**																																			
(16)	attitude toward multimedia learning	0.17^***^	0.14^***^	0.09^*^	0.19^***^	0.23^***^	0.15^***^	0.10^**^	0.16^***^	0.20^***^	0.22^***^	0.31^***^	0.23^***^	0.46^***^	0.48^***^	0.51^***^	(0.89)		
(17)	overall valence of open response^a^	0.16^*^	0.15^*^	0.11	0.07	0.19^**^	0.21^**^	0.16^*^	0.29^**^	0.21^**^	0.30^**^	0.23^**^	0.26^**^	0.37^**^	0.37^**^	0.39^**^	0.50^**^	—	
(18)	appraisal of online learning^a^	0.16^*^	0.11	0.09	0.00	0.17^*^	0.19^*^	0.14	0.24^**^	0.17^*^	0.27^**^	0.24^**^	0.27^**^	0.42^**^	0.40^**^	0.47^**^	0.55^**^	0.86^**^	—
(19)	appraisal of online learning implementation^a^	−0.02	0.13	0.01	0.24	0.32^*^	0.06	0.15	0.26	0.34^*^	0.38^**^	0.15	0.15	0.04	0.33^*^	0.19	0.33^*^	0.68^**^	0.06

*768 < n < 874 for all variables except valence of open response (122 < n < 138), appraisal of online learning (105 < n < 117), and appraisal of online learning implementation (35 < n < 44). Pearson correlations reported except where noted otherwise; numbers in parentheses represent McDonald’s ω*.

a *Kendall’s τ*.

* *p < 0.05;*

** *p < 0.01;*

*** *p < 0.001*.

Attitudes toward multimedia learning also correlated positively with attitudes expressed in open-ended questionnaire responses (0.33 < τ < 0.55). The tone of these statements was more often negative (*n* = 57) than neutral (*n* = 43) or positive (*n* = 37). However, separating these overall evaluations into attitudes toward online teaching *per se* and attitudes toward online learning implementation revealed a slightly different picture. While statements on how universities actually implemented online teaching were mostly negative (negative *n* = 28, neutral *n* = 5, positive *n* = 11), positive and negative statements about online learning in general occurred with roughly equal frequency (negative *n* = 43, neutral *n* = 30, positive *n* = 44).

Due to the small sample size, the error margins for estimating correlations between media use and attitude measures obtained by classifying students’ open comments were quite large. Thus, though these correlations’ direction and magnitude were descriptively quite similar to the relationships found with attitudes toward multimedia learning gathered through the standardized self-report scale, most could not be reliably distinguished from a null correlation. One exception to this general descriptive similarity could be found in students’ appraisals of the quality of current implementation of online learning. Students’ appraisal of implementation seemed to correlate somewhat more strongly with use of audiovisual media and interactive tools before and during the lockdown (0.24 < *r* < 0.38) than with other media use ratings (−0.02 < *r* < 0.26). However, given the large uncertainty in these estimates, this descriptive difference may very well be due to chance.

### Differences Between Online and Offline Learning

Analysis of the 137 open responses revealed issues that were relevant for students during the first lockdown and gave insight into their perceptions of online learning. In their statements, students touched upon a wide range of issues, which can be bundled into four main themes: differences between online and offline learning, conditions for successful online teaching, preferences in how online versus offline instruction are used, and general comments on social and psychological consequences of online learning. A total of 88 participants discussed differences between online and offline learning, including (dis)advantages resulting from that. Fifty-one comments touched upon flexibility in time and space, which was mostly seen as an advantage (42 were positive). Students appreciated that they could adjust their study times to fit their current level of motivation and that they could look through learning materials several times, skip parts they did not find helpful, or do additional research whenever necessary. Video and audio recordings were mentioned as being particularly helpful in this context, since they could be repeatedly watched, stopped, rewound, and watched again. Additionally, participants appreciated saving time and money by not needing to travel to campus. This seemed to be especially attractive for students living far away from their university or under tight financial constraints. Moreover, flexibility in time and space was seen to facilitate the coordination of school with other responsibilities (e.g., job meetings, business trips, medical appointments, and illness), which was especially attractive for part-time students. Only one participant found that distance learning made it more difficult to combine work and school responsibilities. For her, it had been easier when she had a clear structured schedule of courses at the university. Another difficulty in learning outside the university building mentioned by five other students, however, was limited access to specific resources, such as laboratory equipment.

A second characteristic of online teaching mentioned by 36 participants was limited social presence and interaction. This was mainly seen as a disadvantage (34 comments were negative) and often used as an argument for preferring face-to-face teaching. Two participants reported that in online settings, teachers were less able to grasp students’ mood and respond to it, for instance by explaining things more slowly. In addition, students felt that they tended to ask fewer questions, that discussions in courses were rarer and less animated, and that spontaneous debates did not occur after class. Two students found it harder to communicate with their teachers. Another student, however, thought digital media intensified contacts to teachers who took the time to answer. For collaboration in groups, we also found contradicting views: Two students felt it was harder to share thoughts, develop ideas, and solve problems in online teams, while another student experienced online group work as more efficient. According to the comments, social constraints online not only hindered learning progress; they also limited opportunities for getting to know people, sharing personal experiences, staying in touch, developing friendships, and building private and professional networks.

A third difference addressed in the open responses was effectiveness of online teaching compared to classroom teaching. The majority of participants who commented on this aspect (22 of 26) perceived online teaching as less effective. The main reason they gave to explain this was that they had trouble staying concentrated and motivated. Students reported drifting away mentally during online lectures, being distracted, or starting to do other things. This caused them to process content less intensively and to need additional explanations. They noted that the lack of compulsory attendance, combined with a set schedule and challenging deadlines, made it harder to stay motived. Moreover, students experienced interactive methods and practical exercises as less fruitful, which constituted a barrier to critical reflection on and application of theoretical knowledge. As a result,—so their impression—both teachers and students needed to invest more time and effort in order to achieve the same output, and teachers’ didactic competences were even more important than in a face-to-face classroom setting. Only few participants (*n* = 4) felt they learned more during online teaching; this was because they were better able to concentrate at home, experienced teamwork as more efficient, or found it helpful to learn *via* video content.

Based on the differences outlined above, 31 students reflected on the suitability of online teaching for different learning settings. Online learning was described as unsuitable for practical training (e.g., in hospitals or companies), for laboratory exercises, or for acquiring physical skills like learning to play an instrument or sew a wound. This is why some students of medicine, chemistry, automation engineering, music, and art perceived online teaching as inadequate for their discipline. In addition, online teaching was characterized as being less appropriate for courses with interactive and collaborative elements like brainstorming, discussions, or group work. Some students of theology, philosophy, or the social sciences who perceived discourse as crucial to their discipline explained their preference for face-to-face teaching using this argument. In contrast, courses focused on knowledge acquisition, such as traditional lectures, were mentioned as being suitable for online teaching. Two students even preferred online to classroom teaching for theoretical courses in general; others differentiated between specific subjects.

### Conditions for Successful Online Teaching

In the unusual situation of the first COVID-19 lockdown, where distance learning was set up almost overnight, students had a good opportunity to observe how (lack of) existing resources impacted the success of online teaching. Analysis of the 47 student comments dealing with this aspect point to the importance of meeting basic requirements for online instruction at an organizational level. Students reported that they were not able to attend courses because these had been canceled or overlapped in terms of scheduling. Information about when, where (i.e., which communication platform), and in which form courses and exams would take place was missing or unclear. This led to a delay in some students’ studies. Access to adequate learning platforms, video conference tools, and collaboration software (including software that met adequate privacy and data security standards) were missing at some universities, as was sufficient technical equipment for teachers.

A high level of institutional flexibility, innovativeness, and willingness to change combined with established e-learning practices were mentioned as key factors for managing this situation. In addition, teachers’ flexibility and willingness to try new tools and methods were seen as important. Students expressed their desire for teachers to keep them updated, stay in contact, and recognize and consider their needs when designing instruction. Thus, a high level of teacher engagement was named as crucial for successful online teaching, together with digital media skills and didactic competencies.

Digital skills and motivation were also mentioned as prerequisites for students themselves to be able to handle new online tools and maintain self-discipline during online learning, respectively. According to two participants, prior experience with e-learning and a positive attitude toward this type of instruction was helpful. A very basic precondition also named by students is a quiet room equipped with adequate technical resources (e.g., personal laptop or computer, large monitor or second screen, and stable Internet connection); this was not available to all participants.

### Preferences for Online/Offline Teaching Design

Based on their experiences during the lockdown, 65 students expressed preferences for the future of (online) teaching at universities. A question discussed quite often (47 students) was the preferred proportion of online to offline instruction. Most commenters (25) opted for an intelligent mixture of both forms in order to combine their respective advantages. The combination of online and offline elements could be either done at the curricular level (e.g., providing both online and offline courses) or at the course level (e.g., *via* hybrid or blended learning concepts). This, so one of the participants, would prepare students for the increasingly but not fully digitalized working world of the future. Another group of 14 students clearly opted for face-to-face teaching, stating that they would prefer not to attend any online courses at all after the Corona crisis. Nevertheless, additional online elements, such as recordings of class lectures or the possibility to attend courses online in exceptional cases (e.g., illness), were welcomed. In contrast to the group with a clear preference for face-to-face learning, eight other students were quite enthusiastic about online teaching and said they would like to be able to attend major parts of their studies online in the future.

In 20 open responses, we found concrete recommendations for how online and offline teaching could be combined at the course level. These suggestions all served the purpose of letting students choose their preferred modus of teaching. The first recommendation was to hold classes face-to-face but to provide video- or audio recordings of the sessions. This would help students who were unable to attend class at a specific time or day and make it easier to study for exams. A second suggestion was a hybrid setting which would allow students to either attend a course in class or participate live online. One student, however, noted that this scenario required some way of ensuring a minimum number of attendants in class to avoid teachers speaking to an empty room. A third suggestion was to offer online exams as alternatives to face-to-face assessment when the situation allowed, for instance in the case of multiple choice tests or individual oral exams.

Based on what did not work well during lockdown, nine students also gave recommendations for how teachers should structure online courses. Since online sessions were experienced as more exhausting, one suggestion was to have more breaks than in face-to-face class and to limit class length to a maximum of 3 h. Given the constraints in social interaction, students suggested finding new ways of activating students in order to avoid fully teacher-centered lectures. When choosing a tool for online courses, they suggested, teachers should ensure the possibility for video and audio calls, since only written chats are not interactive enough. If a course includes self-directed learning, students should be provided with professional learning material (e.g., lecture notes, audio recordings, and videos), tips for further learning resources (e.g., additional explanations and readings), as well as opportunities for asking questions. Finally, when adapting a course from face-to-face to online, students suggested reducing the amount of content covered.

### Possible Consequences of Online Learning for Individuals and Society

Besides these rather practical considerations, we found several (*n* = 18) more fundamental reflections about what a shift to online teaching at universities could mean for individuals and society in the long term. On the individual level, aspects of health and wellbeing were discussed. These included physical aspects, such as eye strain, muscle tension, and back pain resulting from increased screen time, as well as negative psychological effects, such as overburdening, isolation, loneliness, sadness, depression, aggression, or even suicidal thoughts. One participant, however, identified avoiding infections as a positive health effect of online settings. Concerns on a societal level dealt with the loss of interpersonal encounters and social skills on the one hand, and worries about data security and privacy on the other hand. Moreover, one of the participants expressed concern that education runs the risk of being reduced to knowledge acquisition *via* standardized learning materials, neglecting discourse and diversity of thought as fundamental elements of university learning. Three students, however, also identified a positive environmental effect of online teaching on society, namely, the reduction of car traffic.

## Discussion

Our quantitative results illustrate the dramatic change in the use of media in higher education that occurred during the first Corona lockdown in 2020. Students reported much more intensive use of audiovisual media, e-assessments, and interactive tools during the lockdown than they had experienced before Corona. The results also, however, suggest that this change was not quite as radical as might have been expected. The use of classic digital media like e-mail and digital texts remained stable at high levels; the use of social communication tools remained stable at those high (chat/messenger), medium (social networks and online forums), or low (microblogging) levels that had characterized them before Corona. Though differences between the pre-Corona cluster of “PDF-users” and “digital all-rounders” decreased during lockdown, their usage profiles remained distinct: the very large group of PDF-users continued to use digital media much less intensely than the much smaller group of digital all-rounders, with “video-learners” and “e-examinees” falling somewhere in between. Examining the change in interactive tool use more closely also shows that really only the use of web conferencing skyrocketed during the first Corona lockdown. Interactive tools that had been used fairly intensely before Corona—online office tools and wikis—continued to be used at a slightly higher rate during the lockdown. Rare interactive tools, such as educational games and simulations, remained rarely used. This may have been due to pragmatic reasons, such as the cost and effort involved in developing appropriate educational games or virtual laboratories. It stands to reason that instructors catapulted suddenly into full distance teaching may not have had the time or resources to produce more than rudimentary interactive content. The fact that, among audiovisual media, use of online tutorials increased less than that of audio or video formats is in keeping with this explanation. Many of the comments in the qualitative analysis also suggest that traditional face-to-face learning scenarios (e.g., lectures) were translated 1:1 into online web conferences, with occasional audio or video recordings of those classes but no major adaptations in pedagogy or course structure. Long online sessions without variety or sufficient breaks, lack of interaction and communication, teacher-centered lectures, and insufficient learning materials for self-directed learning were all mentioned by students, showing that even fairly basic pedagogical changes necessary for engaging online instruction were not always implemented. Given the crisis setting, this is understandable. However, it also suggests that, though distance learning brought about a complete change in *where* instruction was carried out, the changes in *how* instruction was structured were much more moderate.

In fact, since one of the most frequent complaints of students was a lack of interaction in online classes, we could argue that the interactive potential of e-learning remained underutilized. It is possible that students simply did not experience the full interactive potential of online instruction because only rudimentary use was made of interactive tools. This is in line with arguments that changes in pedagogy are necessary to facilitate interactive learning (e.g., Webb, 2009) and that effective online teaching requires specific didactic strategies for promoting social interaction (e.g., Kreijns et al., 2003). Though such untapped potential presumably played a substantial role in decreasing the interactivity of online learning during the first Corona lockdown, the inherent social limits of online communication are also likely to have contributed. As long as online communication cannot recreate the full richness of interpersonal contact, feelings of social presence will not be equivalent to face-to-face interaction (e.g., Lowenthal, 2010). In fact, a defining element of the online distance learning situation—independence of place—guarantees that even if new technologies were able to perfectly simulate classroom presence, they could not ensure that students share the same learning context. After class, students would be immediately “transported” back into their own individual environments, and many of the informal opportunities for social interaction that come with the physical limitations of such a shared context (e.g., *ad-hoc* discussions after class and shared breaks) would not occur. The fact that students repeatedly mentioned the disappearance of incidental communication as a clear disadvantage of online learning underlines its subjective relevance.

Despite the limited online interactivity apparent in our data, the open responses also showed the many pragmatic advantages of flexibility in time and place of instruction for students. Without time-consuming journeys to and from campus, students had more time to spend on learning or leisure activities. Online learning was seen as especially attractive for students with other family- or work-related commitments. This is in line with earlier studies touting the sheer convenience of online instruction (e.g., Cole et al., 2014). While this flexibility was seen as challenging in terms of self-discipline and time management, it also allowed students to choose when and how they engaged with course material in accordance with their individual learning needs. Thus, it also promoted self-directed learning.

Overall, the very heterogeneity of results showed that online learning preferences are ultimately individual and depend strongly on students’ specific personal situations as well as the specific content being taught. Though many students reported having concentration and motivation problems, some students were able to learn effectively. Students’ perception of whether online learning was a suitable format depended not only on their personal needs and experiences but also on the content, learning goals, and the relevance of hands-on skills in their own course of studies. Such results bolster Anderson’s (2008) call for flexible use of a variety of instructional media suited to each specific learning context and suggest that context-specific combinations of online and offline instruction are likely to be the most effective course of action. This, however, raises the future research question of which specific learning settings (e.g., face-to-face learning, blended learning, learning with collaborative media, and hybrid learning, …) can best support which specific learning needs and goals, and which individual differences mediate perceptions of the effectiveness of such (online) learning settings. It also raises the practical question of how educational institutions can support teachers in implementing such flexible instructional practices, both in terms of didactic training and in terms of technical support and infrastructure.

Our exploration of student attitudes toward online learning offers some tentative insight into the question of students’ perception of online learning settings. Students’ general attitudes toward multimedia learning showed positive relationships with their media use both before and during the lockdown, as well as a somewhat stronger relationship with their desired media use after Corona. This positive correlation could mean that more intensive media use during the lockdown positively impacted participants’ attitudes toward multimedia. Conversely, it could mean that participants with positive *a priori* multimedia attitudes had actively sought study programs with more infrastructure and opportunities for media use that then carried over into the lockdown. Such students may also have availed themselves of the media accessible through their institutions more thoroughly (e.g., by engaging more intensively with multimedia course materials and using media for informal course-related communication). Alternatively, media use attitudes and experiences might both be driven by an unmeasured third variable, such as universities’ financial resources. Whatever its underlying cause, this association was echoed in the qualitative results. Comparing general multimedia attitudes with participants’ comments at the end of the questionnaire showed that these two evaluations harmonized. This was true both in the sense that students with more positive attitudes also made more positive comments, and in the sense that relationships between appraisals of online learning in the comments and media use were quite similar to the relationships between multimedia attitudes and media use described above. Interestingly, students’ appraisals of the quality of concrete implementation of online learning revealed almost no relationship with their appraisals of online learning in general. This shows that some students differentiated between the potential of online learning *per se* and their current experiences. Nevertheless, both these appraisals tended to correlate positively with attitudes toward multimedia learning, media usage during the first Corona lockdown, and desired usage after—though students’ appraisals of concrete online learning implementation did seem to correlate slightly more strongly with their own use of interactive and audiovisual tools during the lockdown than with their use of other types of media. This could mean that the intensity with which universities provided audiovisual and interactive tools was particularly relevant in shaping students’ appraisals of online learning. Though caution is necessary when speculating about the causal mechanisms responsible for these correlations, our results are certainly compatible with the assumption that attitudes toward online learning stand in a reciprocal relationship with past online learning experiences and are a reasonably strong predictor of future e-learning wishes.

In interpreting these results, several additional limitations should be kept in mind. First, we gathered cross-sectional data in a non-probabilistic sample. This imbues all our statistics with a larger degree of uncertainty (i.e., possible bias) than would be the case for a study with random sampling and mandatory participation. Though we did send invitations to participate in the survey to all Austrian universities, self-selection and school-specific communication policies clearly played a substantial role in determining which schools chose to pass on the survey link to their students, and which of those students chose to participate in the survey. Our additional use of convenience sampling *via* personal contacts also tapped specific social networks, possibly making our sample more homogenous in terms of interests and experiences than a truly random sample would have been. Such homogeneity would tend to make confidence intervals artificially narrow and statistical tests more liberal than suggested by a significance level of 5%. Thus, our results—especially those involving inferential statistics—should be seen as possible starting points for further research, not as solid evidence of population effects.

In addition to the limitations of data gathered from a non-probabilistic sample, we relied on self-reports of behavior and attitudes. Asking students to compare their past with their current media use was particularly cognitively challenging. Estimations of past media use are bound to have been biased by the current situation, though it is unclear whether such a bias can be expected to cause contrast or assimilation effects (i.e., whether high current usage is more likely to make previous usage seem artificially low or to inflate estimates of previous usage to align more closely with current high values). Regardless of how exactly these results are skewed, they should certainly be interpreted as *reports* of media use, and thus, imperfect estimations of actual media use behavior. Nevertheless, comparing these results to self-reported media tool usage gathered before the lockdown (e.g., Persike and Friedrich, 2016; Händel et al., 2020) shows that our participants’ self-reported media use before Corona was quite similar in comparison. We were even able to identify media user types similar to those found by Persike and Friedrich, though we did observe a substantially larger group of “PDF-users.” Overall, however, memory effects do not seem to have negatively influenced the general plausibility of this media usage data.

A third major limitation is the rather narrow scope of collected data. As part of a small, informal study performed in the course of teaching, the questionnaire encompassed only a very small selection of relevant variables based on a limited selection of prior empirical research rather than a broad theoretical foundation. Thus, we are able to offer only a restricted overview of students’ media use and attitudes toward multimedia learning during the first Corona lockdown in 2020, including the themes and experiences they most associated with online learning at this point in time. We did not consider the role of socioeconomic or cultural background, access to adequate “home study” infrastructure, physical impairment, or any of myriad personal and context variables that are likely to have impacted students’ online learning. We also gathered no data about the substantial challenges faced by teachers during this time period, or how organizational resources and support helped shape their online instruction experiences. Because our mixed-methods approach was unplanned, the open responses categorized through qualitative analysis were also limited to short sentences written by a subsample of students with—presumably—particularly strong opinions about online learning. Despite these limitations, we hope that the large and fairly broad sample, high topical relevance, and unique insight into students’ thoughts and experiences during this unprecedented upheaval in educational practice justifies dissemination of these limited but interesting results.

Ultimately, the study shows how online learning is embedded in the organizational context and technical infrastructure of the educational institutions in which it occurs, and how strongly its success is determined by the technological, financial, motivational, and pedagogical resources of the students and teachers who create it. Besides the digital and didactic competencies necessary to adapt instructional scenarios to students’ needs and flexibly integrate appropriate digital tools into engaging online lessons, teachers must bring high levels of motivation and consideration into the virtual classroom. Similarly, students are challenged to maintain the motivation, initiative, and self-regulation necessary for successful self-directed learning. Organizations must offer the necessary IT-infrastructure and support to ensure the functionality and usability of online learning environments. Through the 2020 COVID-19 lockdown, students, teachers, and universities alike were thrown into a highly challenging online learning situation in which these criteria were only imperfectly met. It remains for future research to determine whether the use of interactive tools integrated into holistic pedagogical concepts has become more frequent or varied as the lockdown continues into 2021, and what developments the return to face-to-face instruction will bring. At least some long-lasting changes seem likely. Students have experienced intensive online learning firsthand, including the practical advantages that come with independence of time and place as well as self-directed learning. They have also experienced the interactional limitations of online communication and presumably garnered a new appreciation for the power of face-to-face interaction. The greatest practical and theoretical challenge facing higher education at the moment is determining how to best integrate and leverage the strengths of both forms of learning in a way that not only ensures positive educational outcomes but also meets the changed needs and expectations of organizations, students, and teachers alike.

## Data Availability Statement

The raw data supporting the conclusions of this article will be made available by the authors, without undue reservation.

## Ethics Statement

Ethical review and approval was not required for the study on human participants in accordance with the local legislation and institutional requirements. The patients/participants provided their written informed consent to participate in this study.

## Author Contributions

CK contributed to the concept and design of the study and performed the statistical analysis. CO was responsible for the qualitative data analysis. TJ contributed to the introduction and discussion. All authors contributed to the article and approved the submitted version.

## Conflict of Interest

The authors declare that the research was conducted in the absence of any commercial or financial relationships that could be construed as a potential conflict of interest.

## Publisher’s Note

All claims expressed in this article are solely those of the authors and do not necessarily represent those of their affiliated organizations, or those of the publisher, the editors and the reviewers. Any product that may be evaluated in this article, or claim that may be made by its manufacturer, is not guaranteed or endorsed by the publisher.

## Footnotes

^1^ https://jasp-stats.org/
